# Applications of Augmented Reality for Prehospital Emergency Care: Systematic Review of Randomized Controlled Trials

**DOI:** 10.2196/66222

**Published:** 2025-02-11

**Authors:** Rayan E Harari, Sara L Schulwolf, Paulo Borges, Hamid Salmani, Farhang Hosseini, Shannon K T Bailey, Brian Quach, Eric Nohelty, Sandra Park, Yash Verma, Eric Goralnick, Scott A Goldberg, Hamid Shokoohi, Roger D Dias, Andrew Eyre

**Affiliations:** 1 Department of Emergency Medicine, Mass General Brigham, Harvard Medical School, 10 Vining Street, STRATUS Center for Medical Simulation, Boston, MA, 02453, United States, 1 617-372-1022; 2 Quantitative Musculoskeletal Imaging Group Research, Department of Radiology, Mass General Brigham, Boston, MA, United States; 3 Harvard Data Science Initiative, Harvard Medical School, Boston, MA, United States; 4 University of Connecticut School of Medicine, Farmington, CT, United States; 5 MetaLeaps Inc, Boston, MA, United States; 6 Center for Advanced Medical Learning & Simulation, University of South Florida, Tampa, FL, United States

**Keywords:** prehospital emergency care, emergency medical services, randomized controlled trials, clinical decision support, training, augmented reality, emergency, care, systematic review, BLS, procedures, traumatic injury, survival, prehospital, emergency care, AR, decision-making, educational, education, EMS, database, technology, critical care, basic life support

## Abstract

**Background:**

Delivering high-quality prehospital emergency care remains challenging, especially in resource-limited settings where real-time clinical decision support is limited. Augmented reality (AR) has emerged as a promising health care technology, offering potential solutions to enhance decision-making, care processes, and emergency medical service (EMS) training.

**Objective:**

This systematic review assesses the effectiveness of AR in improving clinical decision-making, care delivery, and educational outcomes for EMS providers.

**Methods:**

We searched databases including PubMed, Cochrane CENTRAL, Web of Science, Institute of Electrical and Electronics Engineers (IEEE), Embase, PsycInfo, and Association for Computing Machinery (ACM). Studies were selected based on their focus on AR in prehospital care. A total of 14 randomized controlled trials were selected from an initial screening of 2081 manuscripts. Included studies focused on AR use by EMS personnel, examining clinical and educational impacts. Data such as study demographics, intervention type, outcomes, and methodologies were extracted using a standardized form. Primary outcomes assessed included clinical task accuracy, response times, and training efficacy. A narrative synthesis was conducted, and bias was evaluated using Cochrane’s risk of bias tool. Improvements in AR-assisted interventions and their limitations were analyzed.

**Results:**

AR significantly improved clinical decision-making accuracy and EMS training outcomes, reducing response times in simulations and real-world applications. However, small sample sizes and challenges in integrating AR into workflows limit the generalizability of the findings.

**Conclusions:**

AR holds promise for transforming prehospital care by enhancing real-time decision-making and EMS training. Future research should address technological integration and scalability to fully realize AR’s potential in EMS.

## Introduction

### Overview

The prehospital setting represents a critical area of emergency medical care. Emergency medical services (EMSs) providers, such as emergency medical technicians, firefighters, and paramedics care for diverse patient populations in variable in highly acute settings; they are often the first to respond to life-threatening scenarios such as traumatic injury or cardiac arrest. Innovations in prehospital care have led to improvement in patient outcomes over the past several decades, including a reduction in early deaths following traumatic injuries and improved survival from out-of-hospital cardiac arrest following early initiation of basic life support (BLS) procedures [1-5]. However, there remain significant challenges to providing high-quality prehospital emergency care, especially in resource-limited settings. Prehospital emergency care literature reports that top research priorities include augmenting the education and training of EMS personnel as well as improving the management of patients with life-threatening conditions such as asthma exacerbation, traumatic brain injury, and cardiac ischemia [6,7]. Further, improving the availability and response quality of medical control physicians for EMS systems has been cited as an additional area of interest [8].

With the need for improvements in both real-time decision support in prehospital care and the education and training of prehospital care providers, researchers have posited the utility of integrating AR into the prehospital setting. AR technologies are tools to superimpose digitally generated 3D and 2D visual information into a user’s environment in real time for display and guidance. Unlike virtual reality, in which a user is completely immersed in a virtual environment that occludes their physical environment, users of AR technologies can interact with both their physical environment and digitally generated images [9].

AR already has significant implications within health care, with AR-based clinical and training modalities beginning to emerge within several medical fields [10-13]. The most well-documented examples come from surgical specialties, which have for years used AR-based equipment as clinical decision support (CDS) and training tools to practice intricate procedures; additionally, many subdisciplines including bariatric surgery, oral-maxillofacial surgery, and neurosurgery use AR-based minimally-invasive robotic procedures [14-19]. Experts have suggested that AR-based CDS tools may prove useful to a variety of prehospital applications, such as providing real-time decision support for patient resuscitation or enhancing BLS education.

To date, there have been few systematic examinations of AR in emergency medicine (EM), with even fewer specifically investigating prehospital emergency medical care. This manuscript thus presents a systematic review of randomized control trials (RCTs) investigating applications of AR in prehospital emergency medical care. Our primary objective is to evaluate the efficacy and effectiveness of AR applications in improving patient outcomes, care processes, and learning outcomes in the prehospital emergency care setting. Our secondary objectives are to identify challenges and limitations for the implementation of AR-based CDS and training tools in prehospital EM and to explore future directions for AR applications in these domains.

## Methods

### Literature Search

A systematic review of the available literature was performed to investigate the effect of AR on prehospital emergency medical care. Eligibility criteria for inclusion in the systematic review included peer-reviewed manuscripts published between 1970 and 2024 (June 10) in English-language journals. A search was conducted of online academic databases including PubMed, CENTRAL, Web of Science, Institute of Electrical and Electronics Engineers (IEEE), Embase, PsycInfo, CINAHL Complete, and Association for Computing Machinery (ACM). Detailed search strategy across databases for identifying studies on AR in prehospital emergency care can be found in Multimedia Appendix 1.

### Full-Text Review

A search of these 8 academic databases yielded 2081 manuscripts for review. Two independent reviewers first screened titles and abstracts to remove duplicates (n=726) as well as manuscripts that were not related to EM (n=1228). A full-text review of 127 studies was conducted by 8 independent researchers to assess their eligibility. Studies were included in full-text screening if a reviewer consensus of 2 reviewers deemed the study eligible. Each study during full-text screening was reviewed by 2 of the 8 reviewers independently and consensus was determined by a third reviewer. Data extraction was conducted independently by 2 reviewers using Covidence software (Veritas Health Innovation), which facilitated the management and review of manuscripts. Each reviewer independently extracted data, including study characteristics, participant demographics, intervention details, and outcome measures. Any discrepancies in the extracted data were resolved through discussion, with a third reviewer stepping in to make the final decision when necessary. No automation tools were used in the data extraction process. The full data extraction form can be seen in Multimedia Appendix 2.

### Criteria for Inclusion

Criteria for inclusion into the final systematic review included full RCT or crossover RCT design; study setting in an EM; and use of wearable, handheld, or projection-based AR in intervention. Studies were included if they investigated the impact of AR on health care professionals or health care students, including emergency responders, paramedics, emergency medical technicians, medics, EM physicians, residents, or fellows, physician assistants, medical and health care students, surgeons, nurses, firefighters, law enforcement officers, or other relevant population (eg, lifeguards, other university students and lay first-responders, or unspecified medical specialties). Studies were also excluded if they were only a description of the technology without learning, performance, or other intervention outcomes.

### Key Data Extracted

Primary outcomes of interest included patient outcomes or clinical performance outcomes such as task completion time, accuracy, number of attempts, and errors. Secondary outcomes included user experience or human factors outcomes such as technology acceptance, workload, stress, and cyber- or simulator-sickness. Key data for analysis was extracted from each of the included manuscripts by 2 independent reviewers using a standardized data extraction form. All data were collected and recorded using Microsoft Excel software. Data collected included study characteristics, participant demographics, AR information, outcome measures, results, and limitations.

In addition to primary outcome measures such as task completion time, procedure accuracy, and protocol compliance, we collected data on several other key variables. These included study characteristics (publication year, country of study, design type, sample size), participant characteristics (professional roles such as first responders, paramedics, medical students; study population size; and whether the setting was civilian or military). Intervention characteristics were also documented, focusing on the type of AR platform used (eg, HoloLens, Vuzix, and Google Glasses) and the intervention context (real-time clinical support or educational training). Secondary outcome measures like user experience, technology acceptance, workload, and the occurrence of simulator sickness were also analyzed. No assumptions were made about missing or unclear data, and any such data were marked as “not reported.”

### Consensus

**Figure 1.**
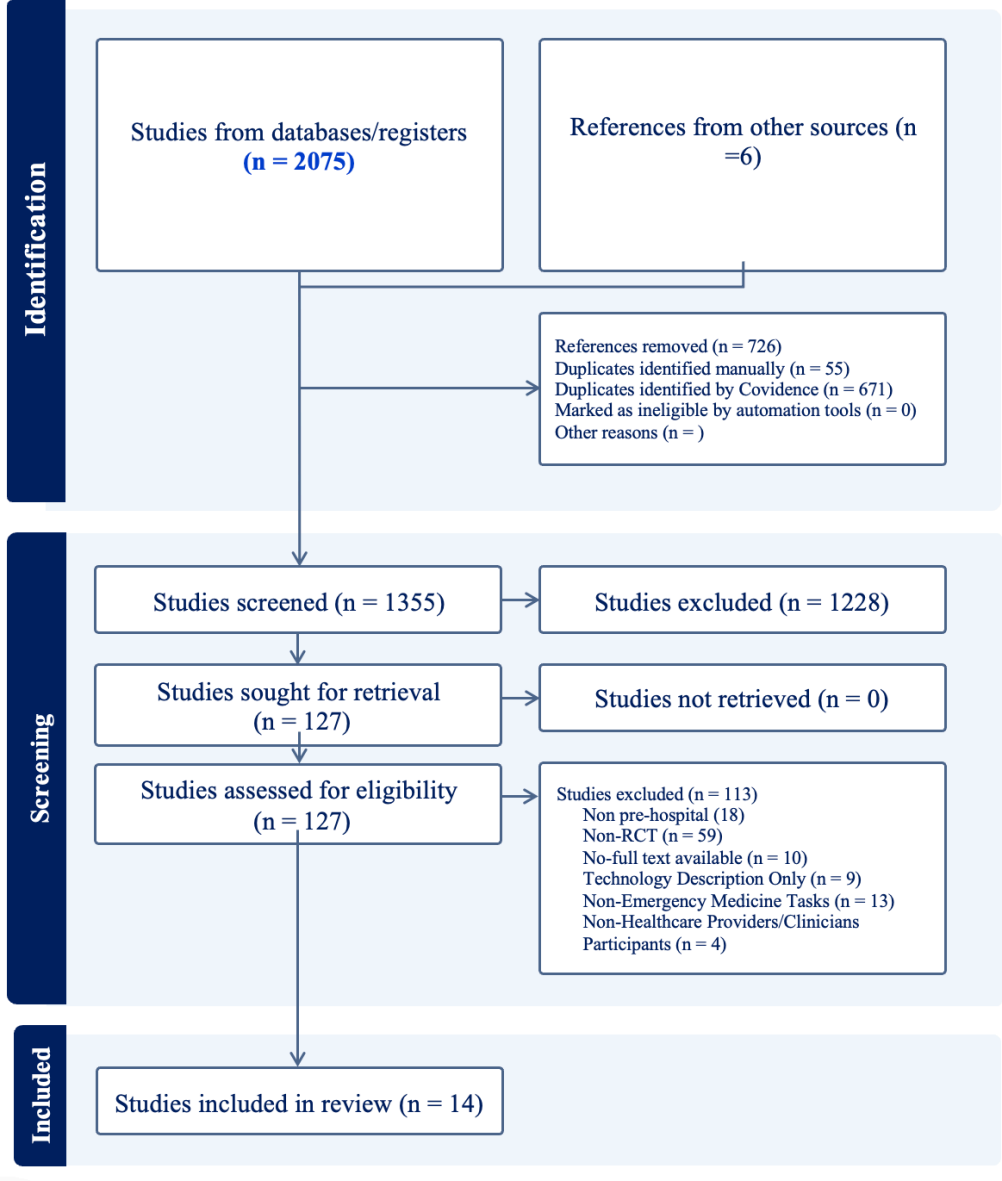
Systematic literature review procedure and the resulting number of relevant papers using PRISMA (Preferred Reporting Items for Systematic Reviews and Meta-Analysis) [21]. RCT: randomized controlled trial.

Consensus between reviewers was tracked via Microsoft Excel spreadsheet and calculated using Cohen κ, with an average of 0.71 (95% CI 0.635‐0.785). The quality and potential bias of the included studies were evaluated on a manuscript level by independent reviewers using Cochrane’s risk of bias tool [20], which can be seen in Multimedia Appendix 3, and reviewed by group consensus. The literature review and evaluation process are detailed in Figure 1. All data were summarized collectively and reported as an aggregate as well as in subgroups including “education and training” and “clinical decision making”. Qualitative and descriptive data were synthesized narratively. The review protocol can be accessed in the Multimedia Appendix 2.

## Results

### Characteristics of Included Studies

Figure 1 presents the review procedure and the resulting number of relevant papers based on PRISMA (Preferred Reporting Items for Systematic Reviews and Meta-Analysis) [21]. The characteristics of the 14 studies included in this systematic review are summarized in Table 1.

**Table 1.** Summary of studies evaluating augmented reality (AR) interventions in prehospital care, including study populations, AR platforms used, primary outcomes, and main findings across various emergency medical scenarios.

First author, publication year	Study population and sample size	AR intervention; platform	Primary outcome measures	Main findings
Rebol et al, 2023 [22]	First responders (n=25)	Real-time assistance for CPR^a^ performance; HoloLens	CPR performance metrics (compression depth and rate)	No significant performance difference between mixed reality and control group
Koutitas et al, 2019 [23]	EMS^b^ cadets (n=30)	Training module for the operation of AmBus systems; HoloLens	Time to task completion and error rate	Significant reduction in task completion time and error rate in AR group
Gruenerbl et al, 2018 [24]	Nursing students (n=50)	CPR training module; Google glasses	CPR performance metrics (compression depth and rate) before and after training	Significant improvement in posttraining performance in AR group
Doswell et al, 2020 [25]	First responders (n=10)	BLS^c^ procedures training module; HoloLens	Time to correct procedure performance	No significant difference in performance time between AR and control group
Collington et al, 2018 [26]	Firefighters (n=10)	BLS procedures training module; Moverio glasses	Performance in simulated trauma scenarios	Significant improvement in self-reported hands-on skills proficiency in AR group
Barcala-Furelos et al, 2023 [27]	Lifeguards (n=38)	Real-time assistance for simulated infant delivery; Vuzix	Performance time and compliance with protocol	Significantly improved protocol adherence in AR group
Follman et al, 2019 [28]	Paramedics (n=31)	Real-time assistance in MCI^d^ triage; ReconJet	Screening time and assessment accuracy	Significant improvement in triage accuracy in AR group
Du et al, 2022 [29]	Medical students (n=20)	Tactical Combat Casualty Care (TCCC) training module; HTC VivePro	Posttest knowledge acquisition	No significant improvement in posttest scores between AR and control groups
Aranda-García et al, 2024 [30]	Health sciences and nursing students (n=60)	CPR and AED^e^ training module; Vuzix	Time to task completion, adherence to BLS protocol, CPR performance	Significantly improved CPR quality and protocol adherence in AR group
Follman et al, 2021 [31]	Non-EM^f^ health care professionals (n=40)	Real-time assistance in MCI triage; ReconJet	Time to triage; triage accuracy	Significantly decreased triage time in non-AR; no difference in accuracy
Hou et al, 2022 [32]	Health care university students (n=27)	CPR training module; HoloLens	CPR performance metrics (compression rate and depth)	No significant performance difference between AR and control groups
Apiratwarakul et al, 2022 [33]	Emergency physicians, nurses, and EMTs^g^ (n=68)	Real-time assistance in MCI casualty detection; HMT-1	Time to completion; accuracy of casualty count in simulated MCI	Significantly decreased time to task completion in AR group, no significant difference in accuracy
Azimi et al, 2018 [34]	EM providers (n=20)	Training in advanced life support procedures; HoloLens	Task performance, task time	No significant difference between AR and control groups
Glick et al, 2021 [35]	Medical students (n=13)	Remote guidance in performing chest thoracotomy; HoloLens	Procedure quality rated by independent observer	Significantly improved procedure quality rating in AR group

a CPR: cardiopulmonary resuscitation.

b EMS: emergency medical service.

c BLS: basic life support.

d MCI: mass casualty incident.

e AED: automated external defibrillator.

f EM: emergency medicine.

g EMT: emergency medical technician.

### Type of Study Design

**Figure 2.**
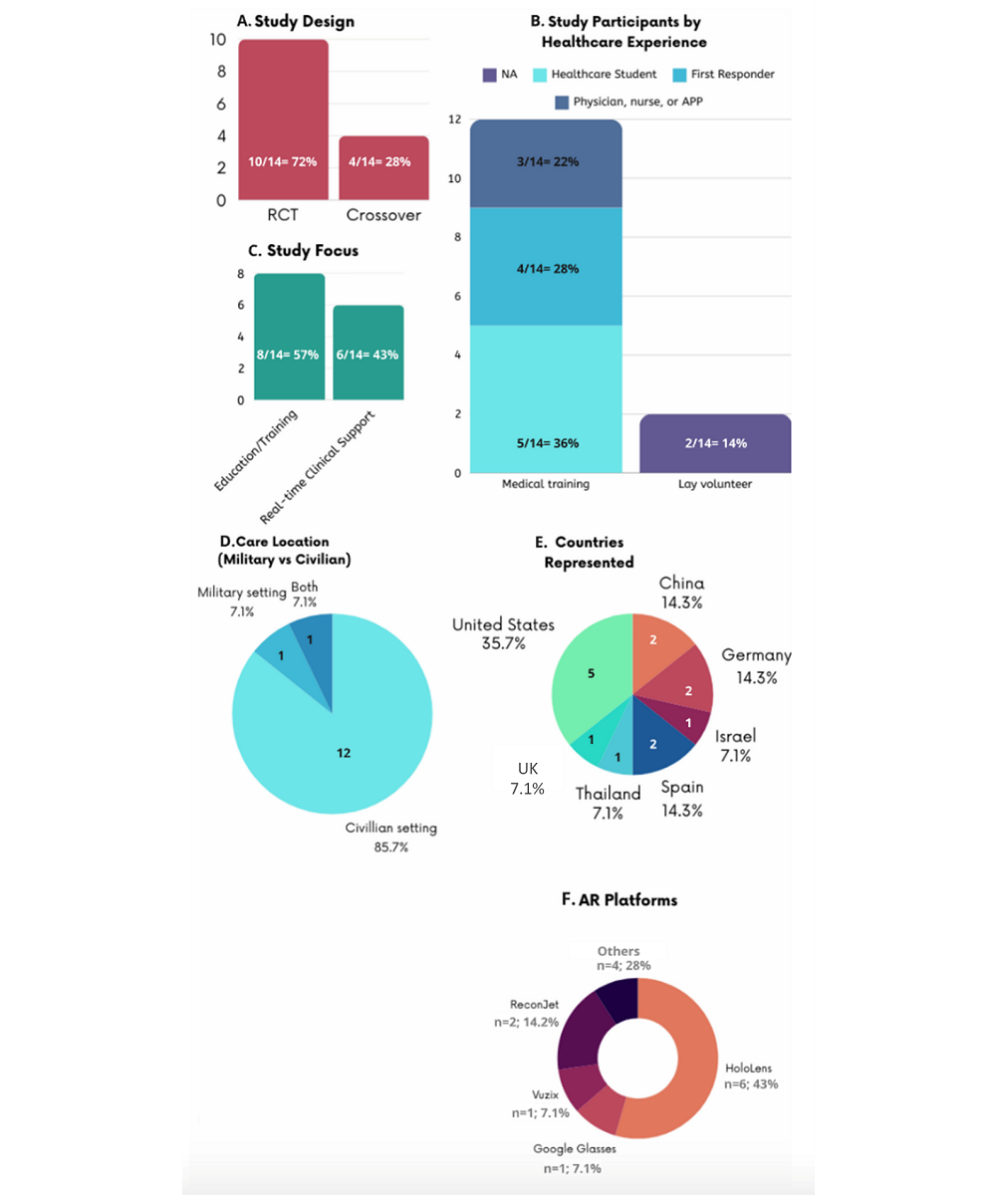
Summary characteristics of 14 included studies.

Figure 2 highlighted the summary-level study characteristics of the 14 studies. Figure 2A and C shows the distribution of studies by study design (Crossover RCT and Full RCT) and their focus areas: real-time decision support, training or education, or both. Full RCTs are the most frequent, with 4 studies focused on training or education and 3 on real-time decision support. Additionally, one study addressed both focus areas. Crossover RCTs primarily focus on training or education (4 studies), with one study focused on real-time decision support.

### Settings and Regions

The 14 studies included a total of 420 participants and were conducted in 7 different countries. A total of 10 (71%) studies were full RCTs while 4 (29%) studies used a crossover design. Overall, 12 (86%) studies were conducted in civilian settings while 1 (7%) study was conducted in a military setting and 1 (7%) study used both military and civilian settings (Figure 2E). Eight (57%) studies used AR for use in task training and education, while the remaining 6 (43%) used AR to provide real-time decision support for clinical scenarios. All 14 (100%) studies used medical simulation rather than real clinical encounters to test their AR interventions.

### Measured Outcomes

While specific outcome measures varied, all studies aimed to compare the efficacy of their AR intervention relative to the current standard of practice. Outcomes examined included time to initiation or completion of desired procedure or intervention (n=5) percentage of correctly informed procedures, procedure quality, or error rate (n=8), and knowledge acquisition (n=1). Overall; 57% (n=8) found statistically significant improvements in their desired outcomes using AR modalities, while 36% (n=5) indicated no significant difference, and 7% (n=1) demonstrated worse performance following AR interventions.

### Type of AR Platforms

All studies used wearable head-mounted displays to deliver their AR intervention (Figure 2F). The most used AR platform across studies was HoloLens goggles (6/14; 43%); other AR platforms used included Vuzix (n=1), Google glasses (n=1), ReconJet (n=2), Epson Moverio (n=1), HTC Vive Pro (n=1), and HMT-1 (n=1). A description of the AR platforms used in the 14 studies is presented in Table 2.

A variety of apps and software platforms were used across the 14 studies; selected novel interventions are highlighted in Table 3.

**Table 2.** Comparison of augmented reality/virtual reality (AR/VR) devices used in prehospital simulations, showing manufacturer, model, release date, price, and key features.

Device	Manufacturer; models and release date; and retail price	Capabilities
**HoloLens**	Microsoft; V2 (2019); US $3500	Eye-tracking, audio and speech command, spatial mapping, MR^a^ capture, Windows connectivity
**Google Glasses**	Google X; Explorer (2019) NOTE: no longer manufactured; US $999-US $1848	Voice command, internet browsing, camera, calendar, android iOS
**Moverio**	Epson; BT 35-e (2018); US $200-US $800	Voice recognition, high definition (HD) display, drone connectivity, remote service, and support
**Vuzix**	Vuzix; M400 (2020); US $1799	Voice recognition, eye-tracking, spatial mapping, iOS and Android compatibility, waterproof
**RealWear**	RealWear; HMT-1 (2018); US $797-US $1500	Voice-activated display, noise cancellation, voice-activated, outdoor-compatible display, water and shock resistant, android and Bluetooth compatible, 20-degree field of view
**ReconJet**	Intel; Smart Glasses (2015); US $699	3-axis sensor, biometric tracking data (heart rate, sleep, etc), GPS, accelerometer, microphones, android iOS compatible, Bluetooth and wifi connectivity
**HTC VivePro**	HTC; VivePro 2.0 (2021); US $699-US $1999	5k resolution, submillimeter tracking capabilities, balanced ergonomic, 120-degree horizontal field of view

a MR: mixed reality.

**Table 3.** Selected augmented reality (AR) apps and software platforms in 14 prehospital included studies.

App	Description	Platform (location)	Manuscript
PRIOR	Android app for technical support in MCI^a^ triage	Tech2Go GMBH Mobile System (Hamburg, Germany)	Follman et al, 2019 [28]
AUDIME	Android app for technical support in MCI triage in the disaster setting	Tech2Go GMBH Mobile System (Hamburg, Germany)	Follman et al, 2021 [31]
AMBUS	App for learning layout of Ambulance Bus Systems	Unity Game Systems (San Francisco, CA)	Koutitas et al, 2019 [23]
Tensor Flow	Artificial intelligence android app for assistance with casualty detection	Google (Mountain View, CA)	Apiratwarakul et al, 2022 [33]
Juxtopia CAMMRAD PREPARE	App for training in BLS^b^ procedures	Juxtopia AR systems (Baltimore, MD)	Collington, 2018 [26]

a MCI: mass casualty incident.

b BLS: basic life support.

### Applications

#### AR as CDS Tools

A total of 6 studies examined AR-based real-time decision support in the prehospital setting. Rebol et al [22] investigated AR-based real-time feedback for adult cardiopulmonary resuscitation (CPR). They found no significant difference in CPR quality in non–health care university students receiving real-time mixed reality–based feedback on performance as compared with students receiving feedback via standard video conference. Barcala-Furelos et al [27] investigated an AR-based intervention aimed at guiding lifeguards assisting in imminent childbirth situations. They found significantly higher adherence to out-of-hospital birth protocols in the AR-intervention group than in the control group (*P*<.05 for all protocol variables). Follmann et al [28] found that real-time AR-based guidance in mass casualty incident (MCI) triage led to a significant improvement in triage accuracy over the control group, which performed triage without AR assistance (*P*=.04). A similar result was found by Follman et al [31], which examined the effect of AR support on MCI triage time and accuracy; they found that triage time was significantly reduced in the control group (*P*<.001) but found no difference in triage accuracy between groups. Apiratwarakul et al [33] employed an AR intervention for assistance in casualty identification; results demonstrated a decreased time to completion of casualty count in the AR group (*P*<.05) but no significant difference in accuracy. Glick et al [35] investigated real-time AR-based guidance for medical students in performing a chest thoracotomy and found that expert rating of procedure quality was significantly improved in the AR group (*P*=.004).

#### AR as Training Tools

A total of 7 studies examined the utility of AR for education and training in the prehospital setting. Two studies (Doswell et al [25] and Collington et al [26]) investigated AR-augmented training for BLS procedures such as Narcan administration and tourniquet application. Doswell et al [25] found no significant difference in procedure time and accuracy between the AR training group and control group; Collington et al [26] showed an increase in self-reported skills proficiency in the AR training group (mean 2.2, SD 1.03) but no significant difference in clinical proficiency. One study [34] examined the efficacy of an AR-based training module on performing advanced life support procedures, including needle chest decompression, direct intravenous placement, and cricothyroidotomy, but found no significant difference in procedure performance between the AR and standard training groups. One study [23] demonstrated that an AR-based training module for familiarization with an AmBus system led to a 10% reduction in time to task completion (involving finding objects on the AmBus) and 34% reduction in errors than the group receiving standard audiovisual-based training. Two studies (Du et al [29] and Follman et al [31]) examined AR-based training for tactical combat casualty care (TCCC) and MCI triage. Du et al [29], which examined TCCC knowledge gain based on pre and posttraining tests, found no significant performance difference between the AR-based training group and the control group.

A total of 3 studies (Gruenerbl et al [24], Aranda-García et al [30], and Hou et al [32]) specifically examined the performance of adult CPR following AR-based training modules. Two of the 3 studies (Gruenerbl et al [24]; Aranda-García et al [30]) found significant improvement in aspects of CPR performance following AR intervention. They demonstrated a significantly improved percentage of time spent performing chest compressions at the correct depth and rate among nursing students receiving AR-based instruction as compared with standard teaching (*P*<.001, *F*=14.85). Aranda-García et al [30] demonstrated significant improvement in the percentage of chest compressions performed with adequate chest recoil (*P*=.008) among health sciences and nursing students receiving AR-based instruction as compared with control; however, they did not find a significant difference in other metrics. Hou found no significant difference in CPR performance (chest compression rate and depth) receiving AR-based training as compared with instructor-led training.

### Risk of Bias Analysis

Risk of bias of studies was assessed via Cochrane’s risk of bias tool, which examined parameters including sampling technique, adequacy of randomization, reliability of outcome measures, and statistical power (Multimedia Appendix 3). Overall, the quality of the included studies was judged to be high. Each of the 14 studies was examined on a manuscript level with consensus reached between 8 independent reviewers. All 14 studies were determined to have a randomized design, with 10 comprising full RCTs and 4 having a crossover design. Most studies were found to have adequate randomization methodology, similar baseline participant characteristics, reliable outcome measures, and a participant dropout rate below 20%. Two of the 14 studies were recorded as lacking sufficient sample size to achieve 80% power with one recorded as “unable to be determined.”

## Discussion

### Principal Findings

This systematic review sought to examine the application of AR to emergency medical care in the prehospital setting, with the primary objective of evaluating the efficacy or effectiveness of AR apps in improving patient outcomes, care processes, and learning outcomes. Of the 14 studies analyzed in this systematic review, the majority demonstrated a significant improvement in desired outcomes with the integration of AR into their workflow, suggesting that AR may have a valuable role to play in enhancing the quality of prehospital care.

### AR as CDS Tools

Studies investigating the utility of AR in providing real-time CDS demonstrated a significant improvement in at least 1 outcome. AR interventions are especially effective in providing real-time decision support for MCI scenarios, enhancing both the accuracy and efficiency of triage procedures and casualty counts. AR-based remote guidance improved procedure quality for fully-trained medical students performing simulated chest thoracotomy procedures, as well as for laypeople responding to simulated childbirth. These results suggest that AR may have an important role to play in improving medical control for EMS, as AR-based feedback and guidance could greatly enhance decision-making for prehospital care providers as compared with traditional audio feedback [36-38]. Results of these studies also suggest that AR may serve a vital purpose in tactical emergency medicine scenarios, including military and law enforcement operations that could benefit from remote guidance in high-acuity scenarios [35,39]. Future research could investigate AR integration into tactical emergency medicine scenarios, such as SWAT team activations.

It is also important to note the potential integration of AR with other emerging technologies, such as artificial intelligence algorithms, which could further enhance decision support by providing predictive analytics and personalized recommendations [13,40,41]. Combining AR with wearable biometric sensors could offer real-time monitoring of vital signs, providing a context-aware decision support system that enhances situational awareness and operational efficiency [10].

### AR as Training Tools

With regards to education and training, 2 of the 4 studies examining the benefit of AR in augmenting CPR training demonstrated significant improvement in CPR quality following AR intervention. These findings suggest that it may be feasible to integrate AR into CPR training. The study by Koutitas et al [23], which examined an AR-based training module for familiarization with AmBus systems also demonstrated improved task completion and enhanced comfort and familiarity with the vehicle in the AR intervention group, suggesting that AR may prove a useful adjunct to EMS companies in training new hires. Notably, some studies, that examined AR intervention in prehospital education and training modules for skills including, CPR, BLS, advanced life support procedures, and TCCC, showed no difference in performance with AR intervention. It is possible that some of these tasks, which involve a significant number of hands-on skills, were more difficult to adapt from in-person instruction to AR-based training. Future research could more thoroughly explore discrepancies in AR-based training modules among various prehospital clinical skills [42]. Furthermore, the scalability of AR training modules offers a significant advantage for widespread training initiatives, allowing consistent and repeatable training experiences across different geographical locations. This scalability is particularly beneficial for remote and underserved areas where access to high-quality training resources is limited.

### Challenges of AR Technology

Overall satisfaction with AR platforms was high across the 14 studies; manuscripts that solicited user feedback found that most participants reported positive perceptions of the technology. Several common concerns emerged from this user feedback. These common concerns are summarized in Table 4.

Of greatest concern was user comfort as well as occasional unpleasant side effects associated with the use of AR. Several manuscripts indicated that wearable interventions, particularly those including headsets, were not compatible with participants who wore prescription eyeglasses. Additionally, some reported participants experiencing side effects after AR use, including dizziness, headache, and nausea. This constellation of adverse effects is collectively known as “cybersickness [43],” and has been demonstrated to impact AR, mixed reality, and virtual reality users, particularly those who are susceptible to motion sickness [44]. Future research into AR should factor cybersickness risk into study design and look to mitigate side effects. Other common concerns included the costs associated with both the purchase and maintenance of AR platforms [45], as well as inconsistent user interface and frequent technological glitches [46]. Addressing these concerns requires a multi-faceted approach [47,48]. Collaborations with manufacturers, health care providers, and end users will be crucial in creating AR systems that are not only effective but also user-friendly and economically viable [9,12]. Additionally, ongoing education and support for users can help mitigate some of the initial discomfort and resistance to new technology [49].

**Table 4.** Summary of common concerns related to augmented reality (AR) use in prehospital care, including user comfort, user interface issues, information technology (IT) challenges, and cost.

Concern	Source
User comfort	Headgear uncomfortable or disruptive to workflow, causes unpleasant side effects (Rebol et al, 2023 [22]; Doswell et al, 2020 [25]; Follman et al, 2019 [28]; Du et al, 2022 [29]; Follman et al, 2021 [31]; Hou et al, 2022 [32]) AR implicated: HoloLens, Google Glass, Moverio
User interface	User interface confusing or difficult to use or requires steep learning curve (Follman et al, 2021 [31]; Glick et al, 2021 [35]) AR implicated: HoloLens, ReconJet
IT issues	Poor battery life, screen glitching, application freezing (Rebol et al, 2023 [22]; Barcala-Furelos et al, 2023 [27]; Aranda-García et al, 2024 [30]; Follman et al, 2021 [31]) AR implicated: HoloLens, ReconJet, Vuzix
Cost	High cost of materials, setup, and maintenance (Du et al, 2022 [29]) AR implicated: HTC VivePro

### Limitations and Future Directions

This systematic review had several limitations. First, many of the included studies were of small sample size. Most studies included under 50 participants, with several included 10 or fewer, which may result in some included studies being underpowered. It is not unusual for studies investigating expensive technologies in potentially cumbersome settings to by necessity include small numbers; however, future research can prioritize adequate sample sizes to ensure robust statistical analyses. Second, our review compared studies with variable outcomes and statistical methodology and thus was not able to examine data in aggregate. A potential next step would be to conduct a meta-analysis of AR interventions in specific emergency prehospital applications, such as CPR training or MCI triage. Third, this review only included studies of AR apps in the prehospital care of adults. Future research will include inquiries into applications of AR for use with pediatric populations. Finally, a main limitation of our search approach was the potential for missed manuscripts due to not features like MeSH headers in PubMed. However, the use of broad search terms across multiple databases helped mitigate this limitation.

### Conclusion

This systematic review shows the promising role of AR technology in enhancing the efficacy of prehospital emergency care. The analyzed studies, involving a total of 14 RCTs demonstrate that AR may enhance clinical decision-making and training modalities within prehospital settings. These improvements are crucial in high-stakes environments where rapid and accurate response is essential. Challenges related to technology integration, cost, and user acceptance remain. Addressing these barriers and conducting further research will be vital for realizing the full potential of AR in prehospital care delivery.

## Supplementary material

10.2196/66222 Multimedia Appendix 1 Detailed search strategy across databases for identifying studies on augmented reality in prehospital emergency care.

10.2196/66222 Multimedia Appendix 2 Systematic review form used for extraction relevant information from included papers.

10.2196/66222 Multimedia Appendix 3 Bias evaluation tool questions.

10.2196/66222 Checklist 1 PRISMA (Preferred Reporting Items for Systematic Reviews and Meta-Analysis) checklist.

## Abbreviations

ACM: Association for Computing Machinery
AR: augmented reality
BLS: basic life support
CDS: clinical decision support
CPR: cardiopulmonary resuscitation
EM: emergency medicine
EMT: emergency medical technician
IEEE: Institute of Electrical and Electronics Engineers
MCI: mass casualty incident
PRISMA: Preferred Reporting Items for Systematic Reviews and Meta-Analysis
RCT: randomized controlled trial
TCCC: tactical combat casualty care
