# Supplementary material for: Applications of Augmented Reality for Prehospital Emergency Care: Systematic Review of Randomized Controlled Trials
Source: JMIR XR Spat Comput. 2025 Feb 11;2:e66222. doi: 10.2196/66222 (PMC13202509; doi:10.2196/66222)
Supplement: Multimedia Appendix 1 [file xr-v2-e66222-s001.docx]

**Appendix 1:** *Detailed search strategy across databases for identifying studies on AR in prehospital emergency care.*

| **Pubmed** | ("Augmented Reality"[Title/Abstract] OR "Holographic"[Title/Abstract] OR "Mixed Reality"[Title/Abstract] OR "Glasses"[Title/Abstract] OR "Smart Eyewear"[Title/Abstract] OR "HoloLens"[Title/Abstract] OR "Google Glass"[Title/Abstract] OR "Smart Glass"[Title/Abstract]) AND ("Emergency*"[Title/Abstract] OR "Emergency Care"[Title/Abstract] OR "Emergency Medicine"[Title/Abstract] OR "Critical Care"[Title/Abstract] OR "Emergency Room"[Title/Abstract] OR "Prehospital Care"[Title/Abstract] OR "Pre-hospital Care"[Title/Abstract] OR "Acute Care"[Title/Abstract] OR "Urgent Care"[Title/Abstract] OR "Emergency Physicians"[Title/Abstract] OR "Triage"[Title/Abstract] OR "Mass Casualty Incidents"[Title/Abstract] OR "Advanced Life Support"[Title/Abstract] OR "Basic Life Support"[Title/Abstract] OR "Ambulance"[Title/Abstract] OR "Emergency Preparedness"[Title/Abstract] OR "Emergency Surgery"[Title/Abstract] OR "Emergency Medical Service"[Title/Abstract] OR "Trauma Surgery"[Title/Abstract] OR "Trauma Room"[Title/Abstract] OR "Trauma Bay"[Title/Abstract] OR "Tele-trauma"[Title/Abstract] OR "Teletrauma"[Title/Abstract] OR "Emergency Department"[Title/Abstract] OR "Paramedics"[Title/Abstract] OR "Emergency Medical Technician"[Title/Abstract] OR "Disaster Medicine"[Title/Abstract] OR "Ambulatory Care"[Title/Abstract] OR "Emergency Services"[Title/Abstract] OR "Trauma Unit"[Title/Abstract] OR "Trauma Hospital"[Title/Abstract] OR "Trauma Center"[Title/Abstract] OR "Polytrauma"[Title/Abstract] OR "Injury Management"[Title/Abstract] OR "Cardiopulmonary Resuscitation"[Title/Abstract] OR "CPR"[Title/Abstract] OR "Traumatic*"[Title/Abstract] OR "Combat Casualty Care"[Title/Abstract] OR "Battlefield Medicine"[Title/Abstract] OR "Combat Trauma"[Title/Abstract] OR "Gunshot"[Title/Abstract] OR "Tactical Medicine"[Title/Abstract]) |
| --- | --- |
| **IEEE** | ("Abstract":"Augmented Reality" OR "Abstract":"Holographic" OR "Abstract":"Mixed Reality" OR "Abstract":"Smart Eyewear" OR "Abstract":"HoloLens" OR "Abstract":"Google Glass" OR "Abstract":"Smart Glass*") AND ("Abstract":"Emergency*" OR "Abstract":"Emergency Care" OR "Abstract":"Emergency Medicine" OR "Abstract":"Critical Care" OR "Abstract":"Emergency Room" OR "Abstract":"Prehospital Care" OR "Abstract":"Pre-hospital Care" OR "Abstract":"Acute Care" OR "Abstract":"Urgent Care" OR "Abstract":"Emergency Physicians" OR "Abstract":"Triage" OR "Abstract":"Mass Casualty Incidents" OR "Abstract":"Advanced Life Support" OR "Abstract":"Basic Life Support" OR "Abstract":"Ambulance" OR "Abstract":"Emergency Preparedness" OR "Abstract":"Emergency Surgery" OR "Abstract":"Emergency Medical Service" OR "Abstract":"Trauma Surgery" OR "Abstract":"Trauma Room" OR "Abstract":"Trauma Bay" OR "Abstract":"Tele-trauma" OR "Abstract":"Teletrauma" OR "Abstract":"Emergency Department" OR "Abstract":"Paramedic" OR "Abstract":"Emergency Medical Technician" OR "Abstract":"Disaster Medicine" OR "Abstract":"Ambulatory Care" OR "Abstract":"Emergency Services" OR "Abstract":"Trauma Unit" OR "Abstract":"Trauma Hospital" OR "Abstract":"Trauma Center" OR "Abstract":"Polytrauma" OR "Abstract":"Injury Management" OR "Abstract":"Cardiopulmonary Resuscitation" OR "Abstract":"CPR" OR "Abstract":"Traumatic*" OR "Abstract":"Combat Casualty Care" OR "Abstract":"Battlefield Medicine" OR "Abstract":"Combat Trauma" OR "Abstract":"Gunshot" OR "Abstract":"Tactical Medicine") |
| **WOS** | AB=(("Augmented Reality" OR "Holographic" OR "Mixed Reality" OR "Smart Eyewear" OR "HoloLens" OR "Google Glass" OR "Smart Glass") AND ("Emergency*" OR "Emergency Care" OR "Emergency Medicine" OR "Critical Care" OR "Emergency Room" OR "Prehospital Care" OR "Pre-hospital Care" OR "Acute Care" OR "Urgent Care" OR "Emergency Physicians" OR "Triage" OR "Mass Casualty Incidents" OR "Advanced Life Support" OR "Basic Life Support" OR "Ambulance" OR "Emergency Preparedness" OR "Emergency Surgery" OR "Emergency Medical Service" OR "Trauma Surgery" OR "Trauma Room" OR "Trauma Bay" OR "Tele-trauma" OR "Teletrauma" OR "Emergency Department" OR "Paramedic" OR "Emergency Medical Technician" OR "Disaster Medicine" OR "Ambulatory Care" OR "Emergency Services" OR "Trauma Unit" OR "Trauma Hospital" OR "Trauma Center" OR "Polytrauma" OR "Injury Management" OR "Cardiopulmonary Resuscitation" OR "CPR" OR "Traumatic*" OR "Combat Casualty Care" OR "Battlefield Medicine" OR "Combat Trauma" OR "Gunshot" OR "Tactical Medicine")) |
| **Embase** | ('Augmented Reality':ab OR 'Holographic':ab OR 'Mixed Reality':ab OR 'Glasses':ab OR 'Smart Eyewear':ab OR 'HoloLens':ab OR 'Google Glass':ab OR 'Smart Glass':ab) AND ('Emergency Care':ab OR 'Emergency Medicine':ab OR 'Critical Care':ab OR 'Emergency Room':ab OR 'Prehospital Care':ab OR 'Pre-hospital Care':ab OR 'Acute Care':ab OR 'Urgent Care':ab OR 'Emergency Physicians':ab OR 'Triage':ab OR 'Mass Casualty Incidents':ab OR 'Advanced Life Support':ab OR 'Basic Life Support':ab OR 'Ambulance':ab OR 'Emergency Preparedness':ab OR 'Emergency Surgery':ab OR 'Emergency Medical Service':ab OR 'Trauma Surgery':ab OR 'Trauma Room':ab OR 'Trauma Bay':ab OR 'Tele-trauma':ab OR 'Teletrauma':ab OR 'Emergency Department':ab OR 'Paramedic':ab OR 'Emergency Medical Technician':ab OR 'Disaster Medicine':ab OR 'Ambulatory Care':ab OR 'Emergency Services':ab OR 'Trauma Unit':ab OR 'Trauma Hospital':ab OR 'Trauma Center':ab OR 'Polytrauma':ab OR 'Injury Management':ab OR 'Cardiopulmonary Resuscitation':ab OR 'CPR':ab OR 'Traumatic*':ab OR 'Combat Casualty Care':ab OR 'Battlefield Medicine':ab OR 'Combat Trauma':ab OR 'Gunshot':ab OR 'Tactical Medicine':ab) |
| **PsyInfo- CINAHL Complete** | AB ( Augmented Reality OR Holographic OR Mixed Reality OR Smart Eyewear OR HoloLens OR Google Glass OR Smart Glass* ) AND AB ( Emergency* OR Emergency Care OR Emergency Medicine OR Critical Care OR Emergency Room OR Prehospital Care OR Pre-hospital Care OR Acute Care OR Urgent Care OR Emergency Physicians OR Triage OR Mass Casualty Incidents OR Advanced Life Support OR Basic Life Support OR Ambulance OR Emergency Preparedness OR Emergency Surgery OR Emergency Medical Service OR Trauma Surgery OR Trauma Room OR Trauma Bay OR Tele-trauma OR Teletrauma OR Emergency Department OR Paramedic OR Emergency Medical Technician OR Disaster Medicine OR Ambulatory Care OR Emergency Services OR Trauma Unit OR Trauma Hospital OR Trauma Center OR Polytrauma OR Injury Management OR Cardiopulmonary Resuscitation OR CPR OR Traumatic* OR Combat Casualty Care OR Battlefield Medicine OR Combat Trauma OR Gunshot OR Tactical Medicine ) |
| **AMC** | [[Abstract: "augmented reality"] OR [Abstract: "holographic"] OR [Abstract: "mixed reality"] OR [Abstract: "smart eyewear"] OR [Abstract: "hololens"] OR [Abstract: "google glass"] OR [Abstract: "smart glass*"]] AND [[Abstract: "emergency*"] OR [Abstract: "emergency care"] OR [Abstract: "emergency medicine"] OR [Abstract: "critical care"] OR [Abstract: "emergency room"] OR [Abstract: "prehospital care"] OR [Abstract: "pre-hospital care"] OR [Abstract: "acute care"] OR [Abstract: "urgent care"] OR [Abstract: "emergency physicians"] OR [Abstract: "triage"] OR [Abstract: "mass casualty incidents"] OR [Abstract: "advanced life support"] OR [Abstract: "basic life support"] OR [Abstract: "ambulance"] OR [Abstract: "emergency preparedness"] OR [Abstract: "emergency surgery"] OR [Abstract: "emergency medical service"] OR [Abstract: "trauma surgery"] OR [Abstract: "trauma room"] OR [Abstract: "trauma bay"] OR [Abstract: "tele-trauma"] OR [Abstract: "teletrauma"] OR [Abstract: "emergency department"] OR [Abstract: "paramedi"] OR [Abstract: "emergency medical technician"] OR [Abstract: "disaster medicine"] OR [Abstract: "ambulatory care"] OR [Abstract: "emergency services"] OR [Abstract: "trauma unit"] OR [Abstract: "trauma hospital"] OR [Abstract: "trauma center"] OR [Abstract: "polytrauma"] OR [Abstract: "injury management"] OR [Abstract: "cardiopulmonary resuscitation"] OR [Abstract: "cpr"] OR [Abstract: "traumatic*"] OR [Abstract: "combat casualty care"] OR [Abstract: "battlefield medicine"] OR [Abstract: "combat trauma"] OR [Abstract: "gunshot"] OR [Abstract: "tactical medicine"]] |
