# Supplementary material for: Applications of Augmented Reality for Prehospital Emergency Care: Systematic Review of Randomized Controlled Trials
Source: JMIR XR Spat Comput. 2025 Feb 11;2:e66222. doi: 10.2196/66222 (PMC13202509; doi:10.2196/66222)
Supplement: Multimedia Appendix 2 [file xr-v2-e66222-s002.docx]

**Appendix 2:** *Systematic review form used for extraction relevant information from included papers.*

| **Section** | **Item** | **Description/Options** |
| --- | --- | --- |
| **Study Identification** | Article title |  |
|  | Authors name |  |
|  | Publication year |  |
|  | Journal |  |
| **Study Characteristics** | Type of RCT Study | - RCT (traditional)  - Crossover RCT  - Other (specify) |
|  | Study Location(s): Country |  |
|  | Study Hospital/Institution Name if available |  |
| **Participant Demographics** | Sample Size |  |
|  | Age | Depends on how they reported:   - Range (e.g., 18-65 years) - Mean (SD) - Median |
|  | Gender/Sex Distribution | Depends on how they reported:   - Percentage (e.g., 60% Male, 40% Female) - Number |
| **Intervention Details** | Type of AR Application | - Wearable AR (e.g., AR glasses)  - Handheld AR (e.g., smartphone, tablet)  - Projection-based AR  - Other (Specify): |
|  | AR Hardware Models | - Name of AR Hardware (models) Used: (e.g, HoloLens 2, Magic Leap, Vuzix, etc).   - Other (Specify): |
|  | Civilian or Military? | - Civilian settings - Military settings |
|  | Emergency Medicine Settings | - Pre-hospital - - ED (Triage) - - ED (ER) - - ED (Observational Unit) - - ED (ICU) - - ED (Triage) - - EM - Home Care / Home Hospital - - Urgent Care - Other (Specify): |
|  | Participants | - EM Physicians (attending - Paramedics - EMT (Emergency Medicine Technician) - Fire Fighter - Law Enforcement officers - Medical Students - Other Healthcare Students - Non-healthcare Students - Other (Specify): |
|  | Purpose of AR Application in Emergency Medicine |  |
|  | Duration of AR Intervention if available | - Time Period (e.g., 30 mins/session, # times a week/month)  - Other (Specify): |
|  | Control Group Summary |  |
| **Outcome Measures** | Clinical Outcomes | - Time to Treatment  - Accuracy of Diagnosis  - Patient Survival Rates  - Patient Satisfaction  - Healthcare Provider Efficiency  - Other (Specify): |
|  | Learning Outcomes | - Knowledge test - Technical skills - Non-technical skills |
|  | Usability/Functionality Measures | - SUS (system usability scale) - Technology acceptance - Heuristics - Interviews - Focus group - Other (specify) |
|  | Cost-Effectiveness |  |
|  | Measurement Tools and Techniques | [List items] |
| **Results** | Key Findings | Summary |
|  | Type of Data Analysis | - Descriptive - Inferential (t-test, ANOVA, Chi-square - Qualitative Analysis - Other (specify) |
|  | Report of statistical analysis/ effect Size | - Statistical significance (p-value, confidence interval, etc.): - Effect size (Cohen's d, Odds Ratio (OR), Odds Ratio (OR): - Area Under Curve (AUC): - Other (specify) |
| **AR Technology (Limitations)** | AR Technology Limitations Noted by Authors |  |
| **Reviewer Notes** | Additional Comments |  |
